# Microstructure and Antimicrobial Properties of Zr-Cu-Ti Thin-Film Metallic Glass Deposited Using High-Power Impulse Magnetron Sputtering

**DOI:** 10.3390/ma15072461

**Published:** 2022-03-27

**Authors:** Jian-Fu Tang, Po-Yuan Huang, Ja-Hon Lin, Ting-Wei Liu, Fu-Chi Yang, Chi-Lung Chang

**Affiliations:** 1 Department of Electro-Optical Engineering, National Taipei University of Technology, Taipei City 106, Taiwan; jftang@ntut.edu.tw (J.-F.T.); jhlin@ntut.edu.tw (J.-H.L.); 2 Department of Materials Engineering, Ming Chi University of Technology, New Taipei City 243, Taiwan; m08188022@mail2.mcut.edu.tw (P.-Y.H.); as65822461@gmail.com (T.-W.L.); 3 Center for Plasma and Thin Film Technologies, Ming Chi University of Technology, New Taipei City 243, Taiwan; fcyang@mail.mcut.edu.tw

**Keywords:** ZrCuTi, nanocrystalline, antimicrobial effect, TFMGs, multilayer

## Abstract

Zr-Cu based thin-film metallic glass (TFMG) has good glass-forming ability and the addition of a third element can create a chaotic system capable of inhibiting the nucleation and growth of crystals. This study focused on TFMGs made with Zr, Cu, and Ti in various compositions deposited via high-impulse magnetron sputtering on silicon and 304 stainless-steel substrates. Detailed analysis was performed on the microstructure and surface characteristics of the resulting coatings. Transmission electron microscopy revealed that the multilayer structure changed to a nanocrystalline structure similar to an amorphous coating. The excellent hydrophobicity of Zr-Cu-Ti TFMGs can be attributed to their ultra-smooth surface without any grain boundaries. The excellent antimicrobial effects can be attributed to a hydrophobic surface resisting cell adhesion and the presence of copper ions, which are lethal to microbes.

## 1. Introduction

Bulk metallic glass (BMG) has been incorporated in golf heads [1], cell phone lens modules, micro gear parts [2], and watches in recent years because of their advantageous properties. There have been considerable advances in preparation processes, alloy systems, and structural modeling [3]; however, researchers have had difficulty scaling up manufacturing. Thin-film metallic glass (TFMG) is increasingly being used as an alternative to conventional BMG, due to its ease of manufacture and low cost. Physical vapor deposition (PVD) methods, such as magnetron sputtering, are widely used in the fabrication of TFMGs [4,5], due to the high deposition rate, rapid quenching (above 10^6^ K/s), excellent microstructure, and precise control over the layer composition. Zr-Cu TFMGs feature high strength, superior corrosion resistance, and biocompatibility. Researchers have also shown that the inclusion of a third element can improve the material characteristics. For instance, the inclusion of Al improves thermal stability [6]; the inclusion of Ta increases the hardness and Young’s modulus [7]; W improves glass transition and crystallization temperatures [8]; Ti improves corrosion resistance, electrochemical stability, and cytotoxicity [9]; and N increases hardness and enhances resistance to oxidation [10]. 304 stainless steel is an alloy material widely used in hospitals and the food industry, due to it being easy to keep clean and disinfect [11]. However, 304 stainless steel has no antibacterial properties. The microorganisms themselves survive on 304 stainless steel, which does not meet the standard of active antimicrobial products. Protective coatings are the effective approach for enhancing the antibacterial properties of 304 stainless steel. For biomedical applications, the metal elements of the coating need to be considered regarding potential toxicity for human health. Titanium (Ti) is non-toxic even in large doses, and does not play any known biological role in the human body [12]. Smieszdk et al. [13] reported that nanoscale TiO_2_ coatings obtained by Atomic Layer Deposition (ALD) have displayed promising pro-osteogenic properties and regulate mitochondrial activity. Zirconium (Zr) metal exhibits the highest biocompatibility of all metals in the body, and Zr compounds are of low toxicity [14]. However, these materials have no antibacterial properties and lead to bacterial infections in clinical applications [15]. Copper (Cu) is another essential trace element present in human and animal tissue [15]. However, excessive Cu amounts in the body have been linked to neurodegenerative diseases including Alzheimer’s, Menkes, and Wilson disease [16]. In order to avoid amounts of Cu ions released and subsequently absorbed by the human body, the Cu content of ZrCuTi coatings was decreased by adding the Ti content in the present study. In addition, Suh et al. [17] discovered that ZrO_2_ thin film on Cu substrate could be obtained using the ALD method.

High-power impulse magnetron sputtering (HiPIMS) is widely used to produce hard coatings, due to the fact that the ionization rate is equivalent to that of a cathodic arc [18,19]. This approach has proven highly effective in producing coatings with a smooth surface [20,21] with a density higher than that of DC magnetron sputtering (DCMS) [22]. Nonetheless, there has been relatively little research on the use of HiPIMS deposition for TFMGs. Zeman et al. [23] reported on the fabrication of Zr-Cu films using a hybrid sputtering system, in which HiPIMS plasma was applied to a Cu target, while the Zr target was operated in direct current (DC) mode. Bönninghoff et al. [24] reported that the relative contribution of metallic film forming ions to the overall ion flux was 13% in DC plasma and 96% in HiPIMS plasma. There is a need for further research on the use of HiPIMS for TFMG deposition, due to the fact that the material properties of the resulting film depend on the action of sputtering ions [25,26].

TFMGs are generally produced using an alloy target with fixed alloy concentration; however, BMG ingots cannot be manufactured in sizes applicable to industrial-scale operations, and the use of alloy targets (i.e., a Cu metal disk on the surface of an Al target) is ill-suited to applications requiring a precise element composition [27]. Thus, it is preferable to use a co-sputtering process involving the use of two or more targets simultaneously [28]. In the current study, we created Zr-Cu-Ti TFMGs via HiPIMS using three large metal targets. Our primary objective was to elucidate the influence of HiPIMS power on the chemical composition of Zr-Cu-Ti TFMGs as well as their microstructure and antimicrobial properties.

## 2. Experimental

**Figure 1:**
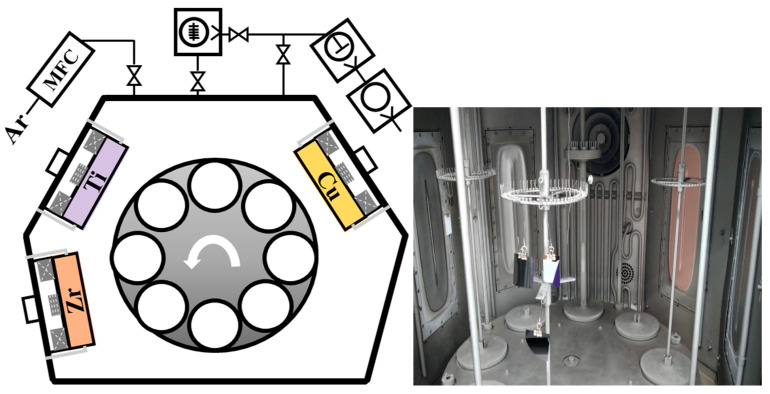
Schematic diagram of experiment apparatus.

TFMGs were deposited on P-type (100) Si and AISI 304 stainless steel substrate using HiPIMS technology. Figure 1 presents a schematic diagram showing the apparatus used in experiments. Three HiPIMS power sources were respectively applied to Zr, Cu, and Ti targets (45.3 × 17 cm^2^). A DC power supply was used for substrate biasing. A substrate holder was used to rotate the samples at 3.5 rpm. The base pressure prior to the experiment was 5 × 10^−4^ Pa. Ar and Ti ion bombardment with a DC bias of −800 V was used to clean the targets and improve adhesion characteristics. Deposition was performed in unipolar mode with a constant pulse on-time of 150 µs (duty cycle of 3%) at a fixed frequency of 200 Hz, while maintaining a target-to-substrate distance of 90 mm and working pressure of 0.4 Pa over a period of 50 min. The output power of the Zr target was fixed at 5 kW and the Ti target power was decreased from 5 to 1 kW while the Cu target power was increased from 0.5 to 1.5 kW. Deposition parameters are listed in Table 1.

The morphologies were evaluated by field emission scanning electron microscopy (FE-SEM, JEOL, JSM-7000F, Tokyo, Japan). The Si substrate was cut in halves for SEM cross-sectional view. Transmission electron microscopy (TEM) images and selected area electron diffraction (SAED) were observed by a TEM instrument (JEOL, JEM-2100 LaB_6_, Tokyo, Japan) with an accelerating voltage of 200 kV. The TEM slices were obtained using a Focused Ion Beam (FIB) technique. A grazing incidence X-ray diffractometer (GIXRD, PANalytical, X’pert, Almelo, The Netherlands) with a Cu Kα radiation was used to analyze the crystallinity of TFMG with an incident angle of 1.5°. A field-emission electron probe microanalyzer (FE-EPMA, JXA-iHP200F, JEOL, Tokyo, Japan) was used to analyze the compositions of the coatings. Thermal properties were measured using a differential scanning calorimetry (DSC, Netzsch 404 F3, Selb, Germany) with a heating rate of 20 K/min under Ar to verify the amorphous nature of the coatings, including the glass transition temperature (T_g_) and crystallization temperature (T_x_). The surface roughness of the stainless-steel substrate and TFMGs was analyzed using an atomic force microscope (AFM, D3100, Bruker, Billerica, MA, USA) and the average contact angle was measured using a contact angle goniometer. The dispensing of a sessile drop of liquid was captured by video camera, whereupon the contact angle of the droplet to the TFMG and stainless-steel substrate was determined using proprietary software. *E. coli* (ATCC 25922) was used to investigate the antimicrobial capability of TFMGs in accordance with the methods outlined in [29]. The antimicrobial performance of the specimens was evaluated via incubation on agar. Bacterial solution (50 μL; 3 × 10^5^ colony-forming units CFU/mL) was applied drop-wise on the surface of as-deposited TFMG and SUS304. The samples were then left to stand at room temperature for 4 h to promote microbe–surface interactions before they were inverted onto Mueller–Hinton agar (MHA, Gibco, Middleton, WI, USA). All samples were incubated individually in an incubator at 37.5 °C for 40 h. The antimicrobial rate was estimated as follows:

(1) $$\mathrm{antimicrobial}\;\mathrm{rate}\;\left(\mathrm{AR}\right)=\frac{N_{0}-N}{N_{0}}\times 100\%$$

where *N*_0_ and *N* respectively indicate the number of viable microbes on a 304 stainless steel sample and TFMGs after the antimicrobial test.

## 3. Results and Discussion

**Figure 2:**
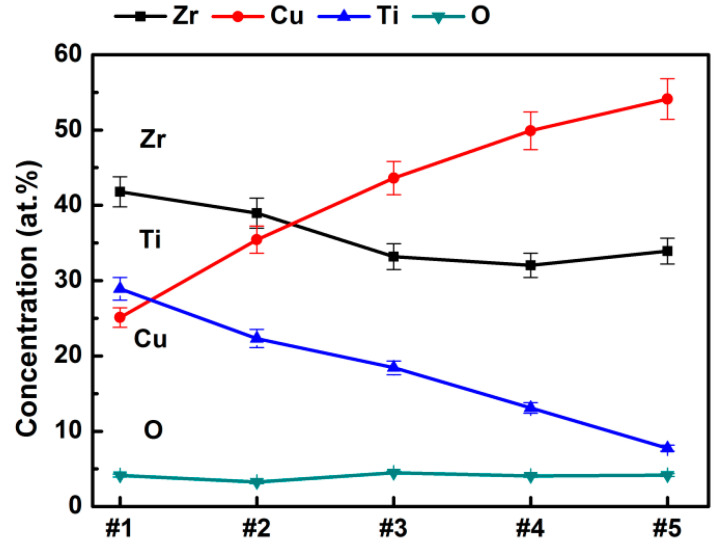
Evolution of chemical composition of Zr-Cu-Ti as a function of sputtering powers.

Figure 2 illustrates variations in the elemental composition of Zr-Cu-Ti films as a function of the sputtering power applied to the metal targets, which evolved monotonically. The content of Cu and Ti element in the Zr-Cu-Ti film was varied precisely by altering the power applied to the Cu and Ti targets. The oxygen content in each coating was less than 5 at.%. Note that the content of Zr in the coatings decreased inversely with Cu content when the Zr target power was fixed. This indicates that the copper atoms replaced zirconium atoms in the material system [30].

**Figure 3:**
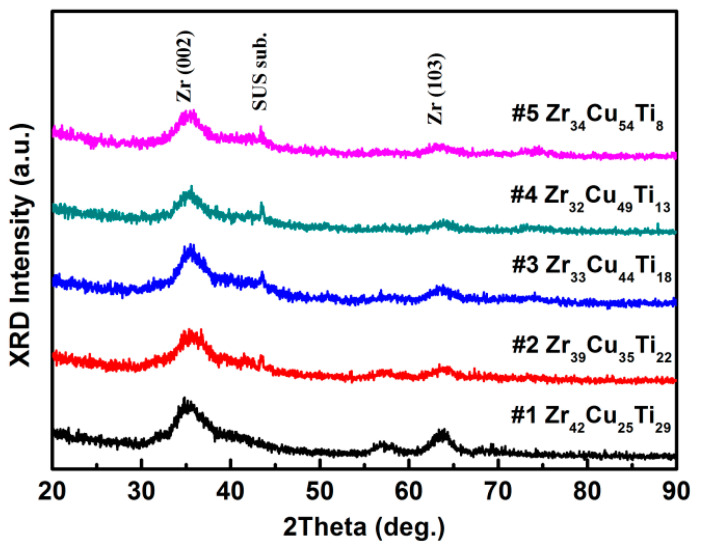
X-ray diffraction patterns of Zr-Cu-Ti coatings of various compositions.

Figure 3 presents XRD patterns of Zr-Cu-Ti films of various compositions. Film #1 presented two broad halo peaks. When the proportion of zirconium in the Zr-Cu-Ti films was reduced, we observed only a broad diffraction hump in the 30–45° 2θ range (i.e., no sharp peaks). Broad peaks in XRD analysis indicate distortions in the lattice parameter due to differences in the size of atoms resulting in decreased crystallinity. A broad peak could indicate an amorphous structure [30], the presence of small crystalline grains [31], or a nanolayer [32]. We therefore examined the structural changes in detail using TEM analysis.

**Figure 4:**
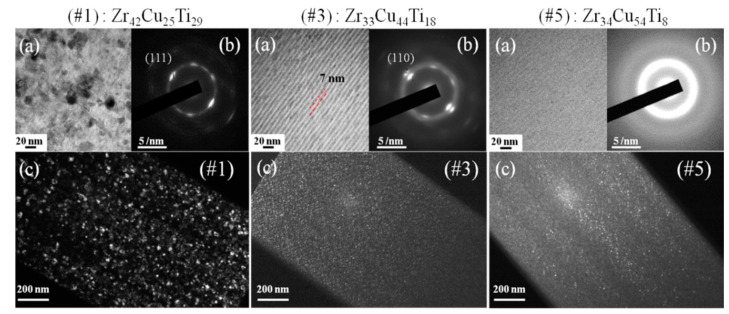
(**a**) Bright-field TEM images, (**b**) SAED, and (**c**) dark-field TEM images of Zr_42_Cu_25_Ti_29_ (#1), Zr_33_Cu_44_Ti_18_ (#3), and Zr_34_Cu_54_Ti_8_ (#5) coatings.

Figure 4 illustrates (a) bright-field (BF) and (c) dark-field (DF) TEM images and (b) SAED of the FIB lamella, showing the microstructure of Zr_42_Cu_25_Ti_29_ (#1), Zr_33_Cu_44_Ti_18_ (#3), and Zr_34_Cu_54_Ti_8_ (#5) coatings produced by varying the output power of Cu and Ti targets. BF TEM images revealed a change in structure to a multi-layer configuration (bi-layer thickness: 7 nm), the boundary of which disappeared as the output power of the Cu target increased (as shown in #1, #3, and #5 sample of Figure 4a). DF TEM images revealed a decrease in grain size from 20 nm to the nanocrystalline scale as well as a reduction in the number of grains (as shown in #1, #3, and #5 sample of Figure 4c). For Figure 4b, the SAED patterns clearly revealed the amorphous structure of thin-film sample #5 and the crystalline structure of thin-film samples #1 and #3. TEM analysis confirmed that the sputtered films were nanocrystalline rather than amorphous, despite the single broad peak in the XRD patterns. The change in crystal structure can be attributed to the high-density plasma and bombardment energy of HiPIMS. The output power of the Zr target was 5 kW, which meets the requirement of HiPIMS discharge [28] leading to the nano-scale grain obtained in sample #1. Increasing the power applied to the Cu target led to the formation of a multilayer structure as the high sputtering yield of copper resulted in most copper ions dominating the growth mechanism [33]. Further increases in the power applied to the Cu target resulted in reduced crystallinity due to differences in atomic size [34].

**Figure 5:**
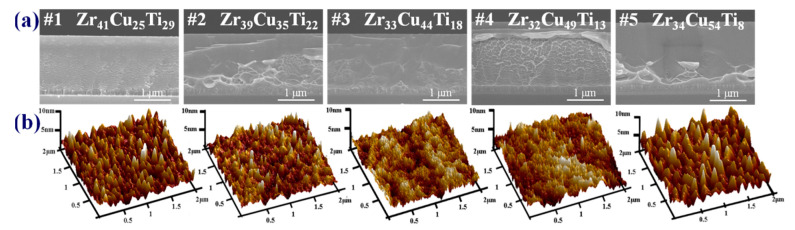
(**a**) Cross-sectional SEM and (**b**) three-dimensional topographical AFM images of Zr-Cu-Ti films of various compositions.

Figure 5a presents cross-sectional SEM images of Zr-Cu-Ti coatings of various compositions. Increasing the input power to the Cu target increased the coating thickness from 1.8 to 2.1 μm, due to an increase in the sputtering yield. Under the Ar ions bombardment with 600 eV kinetic energy conditions, the sputtering yields of the three constituent elements were as follows: Zr (0.7), Cu (2.3), and Ti (0.6) [33]. All of the SEM images revealed shear striations and vein patterns, which are features specific to metallic-glass materials [35,36]. Figure 5b presents a smooth surface profile, measuring 1.3–1.5 nm in Ra, 1.6–1.9 nm in Rq, and 9–11 nm in Rmax. Chang et al. [37] reported that the high surface smoothness of TFMGs can be attributed to a lack of grain boundaries. Despite the appearance of nano-scale grains and multiple layers, the morphological characteristics were similar to those of amorphous glass. Bönninghoff et al. [24] claimed that in HiPIMS samples, an increase in ad-atom mobility due to higher ion energy and flux increases the surface diffusion to beyond that of DC samples, resulting in dynamic smoothing during film growth.

**Figure 6:**
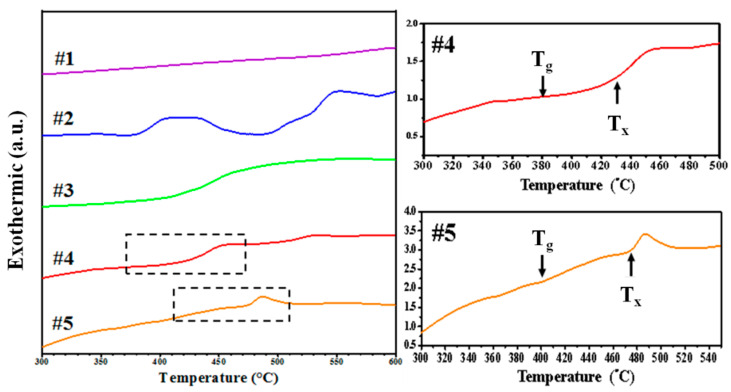
DSC thermograms from Zr-Cu-Ti films of various compositions (#1–#5).

Figure 6 illustrates the differential scanning calorimetry (DSC) results of five coatings. The results were as follows: #4 (glass transition temperature (T_g_) = 651 K and crystallization temperature (T_x_) = 698 K) and #5 (T_g_ = 674 K and T_x_ = 747 K). The supercooled liquid region (i.e., the difference between T_g_ and T_x_) was as follows: #4 (47 K) and #5 (73 K). Clearly, ΔT and glass forming ability increased when the Cu content exceeded 45%. No glass transition or crystallization was observed in the other three coatings, due to the presence of metallic grains in the film [38].

**Figure 7:**
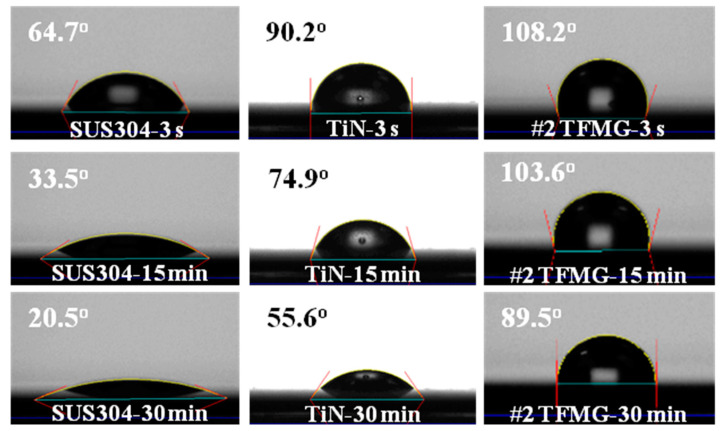
Images of water contact angles on SUS304, TiN, and Zr-Cu-Ti TFMG.

**Figure 8:**
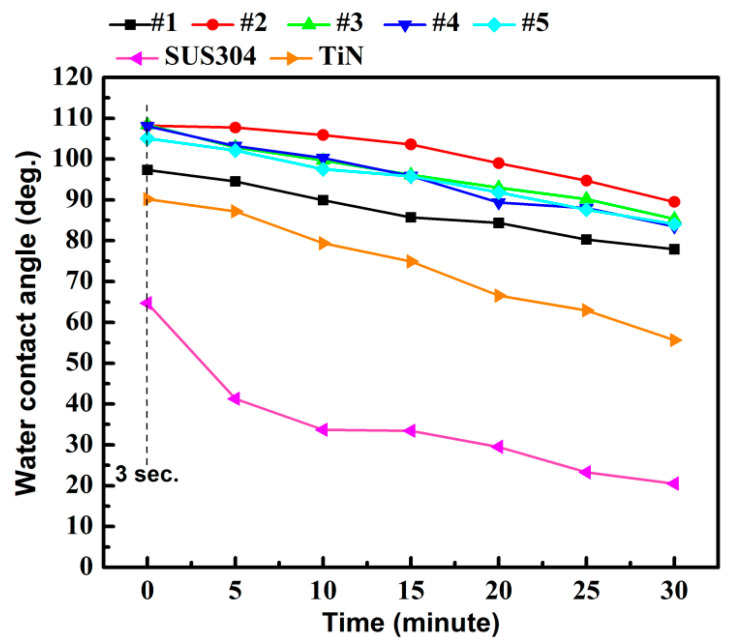
Evolution of contact angle as a function of exposure time under ambient conditions where initial measurements were completed within 3 s.

Most of the tools used in the medical and food sectors are made of SUS304, due to their resistance to corrosion, which contains a high chromium content in the range between 12% and 20% [39]. TiN coatings are also used extensively in the field of biomedicine, thanks to their resistance to wear and high biocompatibility. Therefore, TiN coatings are currently available on the market for knee replacements, particularly aimed at hypersensitive patients [40]. Chiang et al. [41] reported on the relationship between the water contact angle on TFMG-coated surfaces and the presence of bacteria. They claimed that a hydrophobic surface is beneficial to antibacterial efficacy. This can be attributed to the coating having the ability to change the environmental surface and decrease the adherent characteristics of microbes, and ultimately reduce the number of stains involved in infections. As shown in Figure 7, the water contact angle on SUS304 was 64.7°, whereas the contact angle on TiN (90.2°) and Zr-Cu-Ti TFMG (108.2°) exceeded 90 degrees (i.e., distinctly hydrophobic) [25]. Note that on all samples, the contact angle decreased with an increase in measurement time. Figure 8 presents curves indicating water contact angle as a function of time, indicating that metal or metal nitride coatings outperformed the SUS304 substrate in terms of contact angle. By calculating the slope of the curve, we also evaluated the rate at which hydrophobic performance declined. The slopes of the curves were as follows: SUS304 (−1.24), TiN (−1.18), and Zr-Cu-Ti TFMGs (−0.6~−0.8). The long-term hydrophobic properties of the Zr-Cu-Ti TFMGs can be attributed to the amorphous structure (without grain boundaries) and ultra-smooth surface (low roughness) [30].

**Figure 9:**
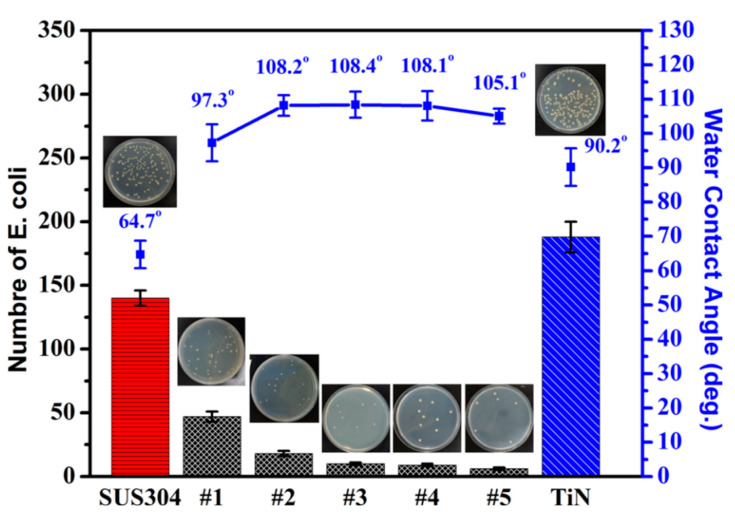
Number of *E. coli* colonies and water contact angle on Zr-Cu-Ti, TiN, and SUS304 samples (insets show photographs of *E. coli* colonies on MHA dishes).

We used the plate counting method to evaluate the antimicrobial efficiency of SUS304 substrate and Zr-Cu-Ti TFMGs of various compositions against *E. coli*. As shown in Figure 9, the uncoated 304 stainless steel substrate and TiN coating exhibited extensive bacterial colonies, far exceeding that on the TFMG samples. The bacterial count was extremely high in MHA dishes, indicating that the antimicrobial ability of TiN coating and stainless steel was poor. On TFMG samples, the number of bacterial colonies decreased from 188 to 6 with a corresponding increase in AR value from 25.4% to 97.6%. The numbers of viable bacterial colonies and AR are summarized in Table 2. The poor AR of TiN coating can be attributed to good biocompatibility [42]. The antimicrobial efficiency of Zr-Cu-Ti TFMGs can be explained as follows: (1) The smooth surface and high hydrophobicity hindered bacterial adhesion, which led to a decrease in the number of *E. coli* bacteria colonies. (2) Cu ions release when bacteria attach to the TFMG surface and interact with the cell membrane. Cells leak solutes to degrade the plastids of DNA, thereby preventing replication [29,30].

## 4. Conclusions

This study investigated the deposition of Zr-Cu-Ti TFMGs via HiPIMS with a focus on the influence of input power to multiple metal targets on the resulting chemical composition, structure, and antimicrobial properties. Compared to the TiN film, the surfaces of the three Zr-Cu-Ti TFMGs structures were far smoother with higher hydrophobicity and a higher antimicrobial rate. Crystals formed due to higher quantities of Zr ions under high bombardment energy. The resulting multilayer structure created an amorphous coating, due to the high sputtering yield of copper, wherein copper ions dominated the growth mechanism. The smooth surface and good hydrophobicity reduced bacterial adhesion, and samples with high Cu content proved lethal to the bacteria, with an antimicrobial rate of 97.6% against *E. coli*, which far exceeded that of SUS304 stainless steel.

## Figures and Tables

**Table 1:** Sample number corresponds to output power of metal targets.

	Samples	#1	#2	#3	#4	#5
Power						
Zr (kW)		5				
Cu (kW)		0.5	0.75	1	1.25	1.5
Ti (kW)		5	4	3	2	1

**Table 2:** Roughness, bacteria number, water contact angle, and antimicrobial rate of SUS304, Zr-Cu-Ti TFMGs, and TiN films.

Sample	Composition	Bacteria Number	AR for *E. coli* (%)	Ra (nm)	Rmax (nm)	WCA (deg.)
SUS304	-	140	44.4	3.24	35	64.72
#1	Zr_42_Cu_25_Ti_29_	47	81.4	1.48	10.31	97.32
#2	Zr_39_Cu_35_Ti_22_	18	92.9	1.25	8.89	108.15
#3	Zr_33_Cu_44_Ti_18_	10	96.0	1.43	9.74	108.40
#4	Zr_32_Cu_49_Ti_13_	9	96.4	1.29	8.97	108.06
#5	Zr_34_Cu_54_Ti_8_	6	97.6	1.49	11.29	105.04
TiN	Ti_49_N_51_	188	25.4	7.44	40.10	90.18

Number of viable bacteria on reference sample was 252 (N_o_).

## Data Availability

Not applicable.
